# Bridging the gap: a cross-sectional study on knowledge and awareness of attention-deficit/hyperactivity disorder among students at a public university

**DOI:** 10.3389/fpubh.2025.1679269

**Published:** 2025-09-29

**Authors:** Geetha Kandasamy, Khalid Orayj, Vanitha Innocent Rani, Asma M. Alshahrani, Tahani S. Alanazi, Amjad Hmlan

**Affiliations:** ^1^Department of Clinical Pharmacy, College of Pharmacy, King Khalid University, Abha, Saudi Arabia; ^2^Department of Community and Psychiatric Nursing, Faculty of Nursing, King Khalid University, Muhayil, Saudi Arabia; ^3^Department of Clinical Pharmacy, College of Pharmacy Shaqra University, Dawadimi, Saudi Arabia; ^4^Department of Clinical Pharmacy, College of Pharmacy King Khalid University, Abha, Saudi Arabia

**Keywords:** Attention-Deficit/Hyperactivity Disorder (ADHD), knowledge, awareness, cross-sectional study, healthcare, non-healthcare

## Abstract

**Background:**

Attention-Deficit/Hyperactivity Disorder (ADHD) is a common neurodevelopmental disorder that affects academic and social performance. Despite increasing mental health awareness, university students including those in healthcare disciplines often have limited understanding of ADHD. This study aimed to assess the levels of ADHD-related knowledge and awareness among students at a public university in Saudi Arabia.

**Methods:**

A cross-sectional study was conducted at King Khalid University, Abha, Saudi Arabia, from February to July 2025. A total of 330 undergraduate students were selected using a non-probability stratified purposive sampling technique. Data were collected using an online questionnaire assessing sociodemographics, ADHD knowledge (9 items), and awareness (11 items). Descriptive statistics and multivariate logistic regression were used to identify associated factors (*p* < 0.05).

**Results:**

Among 330 students, 174 (52.7%) had good knowledge and 117 (35.4%) had good awareness of ADHD. Healthcare students had higher knowledge (78.0% vs. 26.5%), but awareness remained low in both groups (37.5% vs. 33.3%), highlighting gaps that may impede early recognition and support for students with ADHD. Notably, 45.5% of students including 36.3% of healthcare and 54.9% of non-healthcare students believed ADHD could be diagnosed through a blood test, reflecting persistent misconceptions. Lower GPA and reliance on social media were associated with poorer knowledge and awareness, while female gender and advanced academic year predicted better knowledge. These findings underscore the need for targeted educational interventions and evidence-based awareness campaigns to improve ADHD literacy and facilitate timely identification and support.

**Conclusion:**

This study revealed notable gaps in ADHD knowledge and awareness among university students. While healthcare students showed higher theoretical knowledge, practical awareness was low across groups. Better knowledge was associated with higher GPA, academic discipline, and access to professional information. Targeted educational strategies such as workshops, case-based learning, digital resources, and evidence-based campaigns are recommended to enhance ADHD literacy, correct misconceptions, and foster supportive university environments.

## 1 Introduction

Attention-Deficit/Hyperactivity Disorder (ADHD) is a common neurodevelopmental condition characterized by persistent patterns of inattention, hyperactivity, and impulsivity that interfere with daily functioning or development (1). Although traditionally associated with children and adolescents, ADHD frequently persists into adulthood, affecting individuals across the lifespan and posing a significant global public health concern (1). The estimated worldwide prevalence among children and adolescents is approximately 5–10%, with higher rates reported in some populations (1, 2). In Saudi Arabia, a recent meta-analysis reported a pooled prevalence of approximately 12% (3), while US estimates indicate 10–11% of children are diagnosed with ADHD (4). Among specific subgroups in Saudi Arabia, prevalence is higher in student populations, such as 10.9% among medical students in Riyadh (5) and 6.5% among students in Jeddah (6).

The diagnostic criteria for ADHD are outlined in the Diagnostic and Statistical Manual of Mental Disorders, Fifth Edition, Text Revision (DSM-5 TR) by the American Psychiatric Association (7). These criteria require six or more symptoms of inattention and/or hyperactivity–impulsivity for children, or five or more for individuals aged 17 years or older. Symptoms must occur in at least two settings, begin before age 12, cause significant functional impairment, and not be better explained by another disorder (8). While many countries, including Saudi Arabia, also refer to the International Classification of Diseases, 11th Revision (ICD-11), the DSM-5 TR remains widely used in clinical and research settings (9). Core symptoms such as difficulty sustaining attention, excessive fidgeting, impulsivity, and acting without forethought often result in academic and social challenges (10, 11).

ADHD is frequently associated with comorbid conditions, including sleep disorders, learning difficulties, anxiety, and depression, which negatively affect quality of life and academic achievement (12, 13). In educational settings, students with ADHD often struggle with organization, task completion, and consistent performance, resulting in lower grades, reduced retention, and poor test outcomes (14, 15). Knowledge gaps are not limited to ADHD but extend to other neurodevelopmental disorders such as autism spectrum disorder and learning disabilities, where misconceptions and limited awareness among students and educators have been documented (16–19). Collectively, these findings suggest a broader challenge in understanding neurodevelopmental conditions within educational and healthcare contexts, contributing to under-recognition, stigma, and delayed intervention (20).

Despite increasing global awareness, limited data exist on university students’ knowledge and awareness of ADHD, particularly in the Middle East. International studies have highlighted persistent misconceptions, with many students recognizing ADHD as a disorder yet demonstrating gaps in understanding its causes, symptoms, and treatment options (21, 22). Evidence from Saudi Arabia indicates that while awareness exists, accurate knowledge remains insufficient. For instance, a study at Qassim University reported that 83.9% of students were aware of ADHD, yet only 48.2% had adequate knowledge (23). Similarly, another reported that even parents working in healthcare settings showed gaps in knowledge regarding ADHD symptoms, causes, and management, highlighting that misconceptions can persist even among individuals with health-related training (24). Research at King Abdulaziz University and other institutions found that medical students and teachers often lacked comprehensive understanding, although some groups such as male primary school teachers in Riyadh demonstrated comparatively higher awareness levels (16–19, 25).

Several factors influence ADHD knowledge and awareness among university students. Academic discipline and year of study are important, with healthcare and senior students generally showing higher knowledge due to greater exposure. Prior experience with individuals with ADHD, participation in awareness activities, and access to reliable resources also improve understanding. Nevertheless, significant gaps remain across both medical and non-medical students, underscoring the need for targeted educational interventions in Saudi universities (17–19). In Saudi Arabia, public universities represent the largest sector of higher education, enrolling the majority of students from diverse academic and socioeconomic backgrounds. Examining ADHD knowledge and awareness within this setting is therefore particularly relevant, as it provides insights that are broadly representative and generalizable to the wider student population. Moreover, university students especially those in healthcare and education are future professionals who will play a pivotal role in the recognition, diagnosis, and support of individuals with ADHD.

In this context, knowledge and awareness are distinct yet complementary. Knowledge refers to factual and evidence-based understanding of ADHD, including its symptoms, diagnostic criteria, and treatment approaches, whereas awareness encompasses recognition of the condition, perceptions of its prevalence and impact, and attitudes toward affected individuals (16, 21, 22). Awareness is critical because it determines whether students can apply knowledge in real-life situations recognizing ADHD in peers, responding appropriately, and supporting early referral or intervention. Without awareness, even accurate knowledge may not translate into practical recognition, supportive behaviors, or reduced stigma. Consequently, promoting both accurate knowledge and positive awareness is essential for fostering mental health literacy, encouraging timely help-seeking, and reducing barriers to care (19, 20, 25). This study aimed to assess levels of knowledge and awareness of ADHD among university students at a public university in Saudi Arabia. We hypothesized that overall ADHD knowledge and awareness would be suboptimal, with significantly higher levels among healthcare-related students. The findings may help guide targeted educational strategies to enhance mental health literacy across academic disciplines.

## 2 Methods

### 2.1 Study design and sample size

A cross-sectional study was conducted at King Khalid University, Abha, Saudi Arabia, between February and July 2025. A non-probability stratified purposive sampling method was used due to practical constraints and limited access to the full student roster. To ensure adequate representation (53), the Raosoft online sample size calculator was applied as a reference target, assuming a 95% confidence level, a 5% margin of error, and a 50% response distribution. Based on an estimated undergraduate population of 2,000, the calculated minimum sample size was 322 students. A total of 349 students submitted the questionnaire; after excluding 10 incomplete responses and 9 participants who did not provide consent, 330 fully completed responses were included. The reported 94.55% response rate reflects the completion rate among participants who accessed the survey link, rather than a true response rate from a random sample. The findings are therefore representative of the sampled population, with limited generalizability to other universities.

### 2.2 Study population and sampling technique

A non-probability stratified purposive sampling approach was employed to intentionally include key subgroups and ensure adequate representation of students from both healthcare-related and non-healthcare-related colleges. This method was chosen due to practical constraints, including limited access to the full student roster, and to ensure inclusion of participants across different academic years and disciplines. Stratification by academic discipline and year of study helped minimize potential bias (26). Although non-probability sampling may introduce selection bias and limit generalizability, the stratified purposive design mitigates some of these concerns by ensuring representation across key academic streams and levels. This approach is commonly used in educational research when full randomization is not feasible, and when the study aims to explore differences across defined subgroups.

Eight faculties were selected to represent the main academic streams at King Khalid University: medicine, dentistry, pharmacy, applied medical sciences, and nursing (healthcare), and mathematics and English (non-healthcare). These faculties were chosen to provide adequate contrast between healthcare and non-healthcare knowledge bases while considering feasibility and accessibility to students. Within each faculty, participants were recruited from multiple academic years (excluding first-year students, who had not yet received discipline-specific training) to capture variability in exposure to curricula. Recruitment was carried out through institutional platforms, faculty networks, and student communication channels to ensure adequate representation of all targeted subgroups.

### 2.3 Recruitment and data collection

This study targeted currently enrolled students at King Khalid University who were aged 18 years or older and willing to participate. Individuals younger than 18 years and non-university students were considered non-inclusion criteria, as they did not meet the basic eligibility requirements for participation. First-year students were excluded because they primarily study general courses such as English, Islamic studies, and basic sciences, which are common across all programs. They do not yet receive discipline-specific training that could influence their understanding of ADHD. Excluding first-year students ensured that participants had sufficient exposure to their respective academic curricula, allowing for a more accurate assessment of ADHD knowledge and awareness among students who had progressed in their programs.

Other exclusion criteria included incomplete responses and lack of informed consent. Data collection was conducted using a structured, self-administered online questionnaire developed and hosted on Google Forms. The questionnaire was administered in English, the primary language of instruction at the university. The survey link was distributed via multiple university-approved communication channels, including WhatsApp groups, social media platforms, and faculty members prior to lectures. At the beginning of the survey, participants were presented with a detailed informed consent statement explaining the purpose of the study, confidentiality of responses, and the voluntary nature of participation. Only participants who provided affirmative consent were allowed to proceed. To ensure data integrity and prevent duplicate responses, Google Forms was configured to allow only one submission per user through institutional email verification and IP address restriction. No personal identifiers were collected, and responses remained anonymized for analysis. Additionally, incomplete responses and responses from individuals who did not provide consent were excluded from the final analysis.

### 2.4 Study tool

A structured, self-administered questionnaire was used for data collection, comprising three sections. Section 1 captured sociodemographic details, including participants’ age, gender, year of study, department, marital status, Grade Point Average (GPA), and sources of information about ADHD. GPA is a standardized measure of academic performance, typically ranging from 0 to 5, with 0 representing the lowest possible performance and 5 indicating excellent performance. Section 2 assessed participants’ knowledge of ADHD through nine items developed based on a review of relevant literature and previously validated tools (19, 23, 27). This section included nine items (Q1–Q9) covering awareness of ADHD terminology, diagnostic criteria, types, symptom duration, reporting sources, academic impact, comorbidities, diagnostic methods, and treatment approaches. Section 3 evaluated awareness of ADHD using eleven items focused on common ADHD symptoms, aligned with DSM-5 TR (8), and covering the two core domains: inattention and hyperactivity–impulsivity. The items (Q10–Q20) asked participants to indicate whether children often exhibited behaviors such as overlooking details, difficulty maintaining attention, excessive talking, organizational challenges, hyperactive movements, distractibility, lack of social engagement, impulsive responses, acting without clear initiative, and difficulty waiting or being patient. Items in Section 3 were categorized according to the two primary ADHD symptom domains defined in the DSM-5 TR: inattention (attention deficit) and hyperactivity-impulsivity. This approach was adopted to assess participants’ awareness across the two core dimensions of ADHD, rather than evaluating each item solely as correct or incorrect. Grouping items by symptom domain allows comparison of recognition of inattention versus hyperactivity-impulsivity symptoms between healthcare and non-healthcare students. The questionnaire was adapted from previously validated instruments (19, 23, 27) and modified to align with the objectives of this study and the cultural context of Saudi university students. Content validity was ensured through expert review by professionals in psychology and public health, who evaluated item relevance, clarity, and cultural appropriateness. Items were revised based on expert feedback to ensure comprehension and suitability for this population. Although English is the medium of instruction at the university, some students may have had limited proficiency, which could have affected comprehension of questionnaire items and introduced response bias. The original instruments from which it was adapted had demonstrated good validity and reliability (Cronbach’s alpha ranging from 0.75–0.85) (19, 23, 27).

### 2.5 Pilot study

A pilot study was conducted prior to the main data collection phase to assess the clarity, validity, relevance, and reliability of the questionnaire. Sixty students, 30 from healthcare students and 30 from non-healthcare students participated in the pilot study. Based on their feedback, minor revisions were made to improve item clarity and comprehension. Responses from the pilot group were excluded from the final analysis to avoid bias. The pilot study also helped identify potential comprehension difficulties for students with weaker English proficiency, and minor modifications were made to ensure all items were clearly understood. Content validity was ensured through expert review by professionals in psychology and public health, who evaluated item relevance, clarity, and cultural appropriateness. Reliability analysis demonstrated good internal consistency, with Cronbach’s alpha values of 0.72 for the knowledge section and 0.84 for the awareness section, confirming that the questionnaire was reliable and appropriate for use in this population.

### 2.6 Scorings

A total of 20 questions were used to assess participants’ knowledge and awareness of Attention-Deficit/Hyperactivity Disorder (ADHD). Each correct response was given a score of 1, while incorrect responses were scored as 0. The knowledge domain comprised 9 questions (Q1–Q9). Participants scoring ≥6 (≥60%) were classified as having good knowledge, while those scoring below 6 were considered to have poor knowledge. The awareness domain included 11 questions (Q10–Q20), focusing on symptoms of inattention and hyperactivity-impulsivity. A score of ≥7 (≥60%) indicated good awareness, whereas scores below 7 indicated poor awareness. The 60% threshold was selected based on ADHD-specific literature (23) and aligns with previously validated ADHD knowledge and awareness instruments.

### 2.7 Ethical approval

This study was approved by the Ethics Committee of the College of Pharmacy, King Khalid University (Approval No: ECM#2025-107). The first page of the survey included an informed consent statement, outlining the study’s objectives, the voluntary nature of participation, and assurances of confidentiality. Participants were informed that their decision to decline or withdraw from the study at any point would not result in any negative consequences. Although institutional email verification and IP restrictions were used to prevent duplicate submissions, no identifiable personal information was collected, ensuring that all responses remained anonymous. The study was conducted in accordance with the ethical principles outlined in the Declaration of Helsinki.

### 2.8 Statistical analysis

Statistical analysis was conducted using Microsoft Excel and IBM SPSS Statistics software. Descriptive statistics, including frequencies and percentages, were used to summarize categorical variables. The chi-square test was applied to assess associations between sociodemographic variables and ADHD knowledge and awareness. The association between independent variables (age, gender, year of study, department, and GPA) and levels of ADHD knowledge and awareness among healthcare and non-healthcare students was assessed using multivariate logistic regression analysis. The goodness of fit for the logistic regression model was evaluated using the Hosmer-Lemeshow test. A *p* < 0.05 was considered statistically significant.

## 3 Results

Among the 330 university students surveyed, the majority were aged 21–24 years (184; 55.8%), predominantly female (208; 63%), and mostly single (305; 92.4%). Most participants were in their third (104; 31.5%) or fourth (99; 30.0%) year of study, with nearly one-third reporting a GPA above 4.75 (96; 29.1%). Among healthcare students (*n* = 168), primary sources of ADHD information were healthcare providers (55; 32.7%) and academic sources such as books and journals (40; 23.8%), while non-healthcare students (n = 162) primarily relied on internet and social media (78; 48.1%) and family or friends (30; 18.5%) (Table 1). No significant differences were observed between healthcare and non-healthcare students regarding age, gender, year of education, marital status, or GPA, but sources of information differed significantly (*χ*^2^ = 53.164, *p* < 0.001).

**Table 1:** Distribution of sociodemographic variables and ADHD information sources among healthcare and non-healthcare university students.

Variables	Categories	Healthcare (*n* = 168)%	Non-healthcare (*n* = 162)%	Total (*n* = 330)	*χ*^2^ value	*p* value
Age	18–20 years	64 (38.1)	66 (40.7)	130 (39.4)	0.367	0.832
	21–24 years	95 (56.5)	89 (54.9)	184 (55.8)		
	25 years and above	9 (5.4)	7 (4.3)	16 (4.8)		
Gender	Male	66 (39.3%)	56 (34.6%)	122 (37.0%)	0.598	0.439
	Female	102 (60.7%)	106 (65.4%)	208 (63.0%)		
Year of Education	Second year	30 (17.9%)	31 (19.1%)	61 (18.5%)	5.081	0.165
	Third Year	58 (34.5%)	46 (28.4%)	104 (31.5%)		
	Fourth Year	54 (32.1%)	45 (27.8%)	99 (30.0%)		
	Fifth Year	26 (15.5%)	40 (24.7%)	66 (20.0%)		
Marital Status	Single	156 (92.9%)	149 (92.0%)	305 (92.4%)	0.091	0.762
	Married/Divorced	12 (7.1%)	13 (8.0%)	25 (7.6%)		
GPA	More than 4.75	54 (32.1%)	42 (25.9%)	96 (29.1%)	3.738	0.291
	>4.5 to ≤ 4.75	55 (32.7%)	47 (29.0%)	102 (30.9%)		
	>4.25 to ≤ 4.5	38 (22.6%)	44 (27.2%)	82 (24.8%)		
	Less than or equal to 4.25	21 (12.5%)	29 (17.9%)	50 (15.2%)		
Sources of information among students aware of ADHD	Healthcare providers	55 (32.7)	12 (7.4)	67 (20.3)	53.164	0.00001*
	Through internet/social media	40 (23.8)	78 (48.1)	118 (35.8)		
	Family members/Friends	18 (10.7)	30 (18.5)	48 (14.5)		
	Television/Newspaper	15 (8.9)	24 (14.8)	39 (11.8)		
	Books/Journals/University studies	40 (23.8)	18 (11.1)	58 (17.6)		

*χ^2^ = chi sqaure.

**Figure 1:**
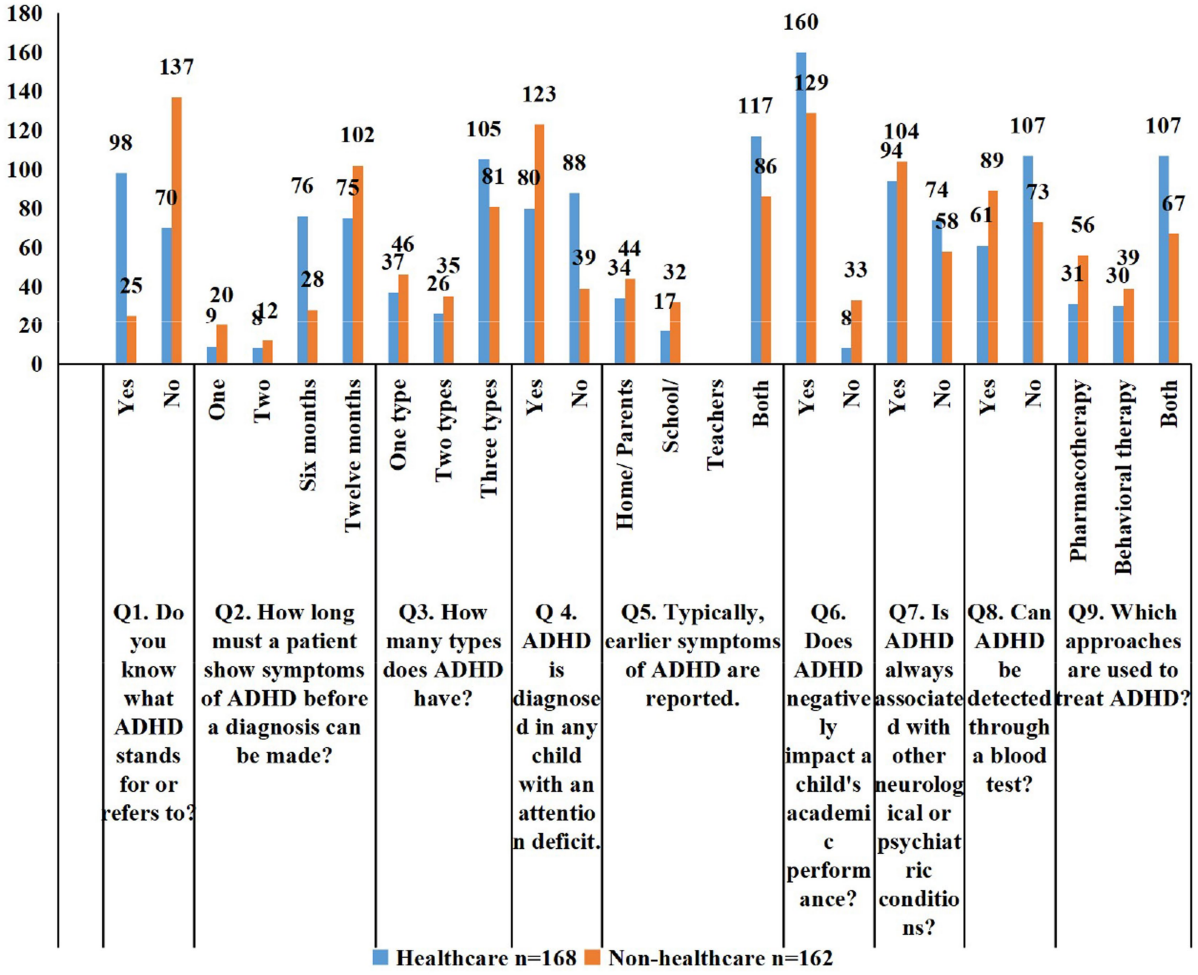
Comparison of knowledge regarding ADHD diagnosis, symptoms, and treatment among healthcare and non-healthcare students.

Overall ADHD knowledge among the 330 students was moderate (Table 2). Only 123 (37.3%) correctly identified the acronym, 104 (31.5%) were aware of the 6-month diagnostic duration, and 186 (56.4%) recognized the three ADHD types. Notably, 203 students (61.5%) incorrectly believed that any child with attention deficits could be diagnosed with ADHD, and 150 (45.5%) thought it could be diagnosed via a blood test, highlighting critical misconceptions. Most students (289; 87.6%) acknowledged the academic impact, and 174 (52.7%) correctly identified combined pharmacotherapy and behavioral therapy as the most effective treatment. Healthcare students demonstrated significantly higher knowledge than non-healthcare students across most domains, including recognition of the acronym (98; 58.3% vs. 25; 15.4%), diagnostic duration (112; 66.7% vs. 65; 40.1%), misconceptions about attention deficits (78; 46.4% vs. 126; 77.8%), blood test misconceptions (61; 36.3% vs. 89; 54.9%), academic impact (160; 95.2% vs. 129; 79.6%), identification of symptom reporters (118; 70.2% vs. 85; 52.5%), and treatment approach (107; 63.7% vs. 67; 41.4%). Awareness of ADHD types (111; 66.1% vs. 75; 46.3%) and association with other neurological or psychiatric conditions (106; 63.1% vs. 92; 56.8%) did not differ significantly between groups (Figure 1).

**Table 2:** Comparison of knowledge regarding ADHD diagnosis, symptoms, and treatment among healthcare and non-healthcare students.

Questions	Response	Healthcare *n* = 168 (%)	Non-healthcare *n* = 162 (%)	Total *n* (%) 330	*χ*^2^ value	*p* value
Q1. Do you know what ADHD stands for or refers to?	Yes	98 (58.3%)	25 (15.4%)	123 (37.3%)	64.923	0.00001*
	No	70 (41.7%)	137 (84.6%)	207 (62.7%)		
Q2. How long must a patient show symptoms of ADHD before a diagnosis can be made?	One	9 (5.4%)	20 (12.3%)	29 (8.8%)	31.146	0.00001*
	Two	8 (4.8%)	12 (7.4%)	20 (6.1%)		
	Six months	76 (45.2%)	28 (17.3%)	104 (31.5%)		
	Twelve months	75 (44.6%)	102 (63.0%)	177 (53.6%)		
Q3. How many types does ADHD have?	One type	37 (22.0)	46 (28.4)	83 (25.2)	5.293	0.070
	Two types	26 (15.5)	35 (21.6)	61 (18.5)		
	Three types	105 (62.5)	81 (50.0)	186 (56.4)		
Q 4. ADHD is diagnosed in any child with an attention deficit.	Yes	80 (47.6%)	123 (75.9%)	203 (61.5%)	27.914	0.00001*
	No	88 (52.4%)	39 (24.1%)	127 (38.5%)		
Q5. Typically, earlier symptoms of ADHD are reported.	Home/ Parents	34 (20.2%)	44 (27.2%)	78 (23.6%)	10.502	0.005*
	School/Teachers	17 (10.1%)	32 (19.8%)	49 (14.9%)		
	Both	117 (69.6%)	86 (53.1%)	203 (61.5%)		
Q6. Does ADHD negatively impact a child’s academic performance?	Yes	160 (95.2%)	129 (79.6%)	289 (87.6%)	18.466	0.00001*
	No	8 (4.8%)	33 (20.4%)	41 (12.4%)		
Q7. Is ADHD always associated with other neurological or psychiatric conditions?	Yes	94 (56.0%)	104 (64.2%)	198 (60.0%)	2.336	0.1264
	No	74 (44.0%)	58 (35.8%)	132 (40.0%)		
Q8. Can ADHD be detected through a blood test?	Yes	61 (36.3%)	89 (54.9%)	150 (45.5%)	11.543	0.0006*
	No	107 (63.7%)	73 (45.1%)	180 (54.5%)		
Q9. Which approaches are used to treat ADHD?	Pharmacotherapy	31 (18.5)	56 (34.6)	87 (26.4)	17.449	0.0001*
	Behavioral therapy	30 (17.9)	39 (24.1)	69 (20.9)		
	Both	107 (63.7)	67 (41.4)	174 (52.7)		

*χ^2^ = chi sqaure.

**Figure 2:**
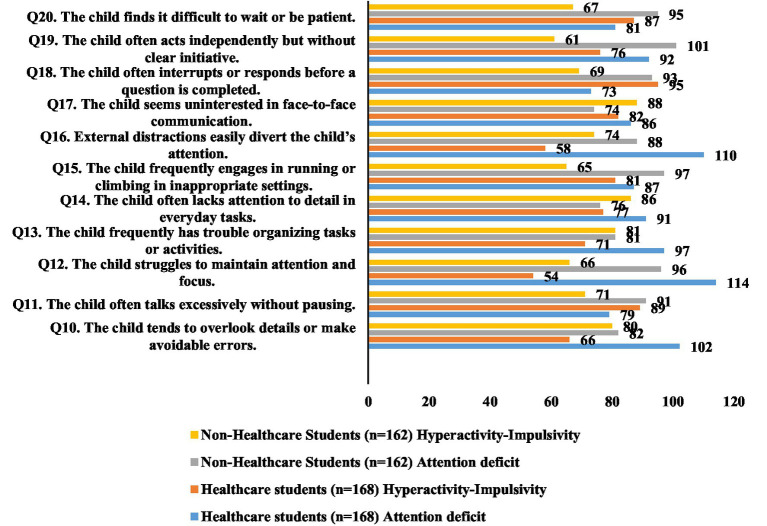
Comparative awareness of inattention and hyperactivity–impulsivity symptoms of ADHD among healthcare and non-healthcare students at a public university.

Table 3 compares healthcare and non-healthcare students’ awareness of inattention and hyperactivity-impulsivity symptoms. Among healthcare students, awareness of inattention symptoms was generally high, with 102 (60.7%) recognizing that children often overlook details and 114 (67.9%) identifying difficulty maintaining attention. A significant difference was observed for “external distractions easily divert attention,” with 110 (65.5%) of healthcare students aware versus 88 (54.3%) of non-healthcare students (*χ*^2^ = 4.276, *p* = 0.038). Non-healthcare students demonstrated higher awareness for some hyperactivity-impulsivity symptoms; for example, 93 (57.4%) correctly identified that children often interrupt or respond before a question is completed, compared to 73 (43.5%) of healthcare students (*χ*^2^ = 6.424, *p* = 0.011). Other symptoms, including difficulty waiting, excessive talking, organizing tasks, and acting independently without clear initiative, showed differences that were not statistically significant (Figure 2).

**Table 3:** Comparative awareness of inattention and hyperactivity–impulsivity symptoms of ADHD among healthcare and non-healthcare students at a public university.

Questions	Healthcare students (*n* = 168)		Non-Healthcare Students (*n* = 162)		*χ*^2^ value	*p* value	Total (*n* = 330)	
	Attention deficit n (%)	Hyperactivity- Impulsivity n (%)	Attention deficit n (%)	Hyperactivity- Impulsivity n (%)			Attention deficit n (%)	Hyperactivity- Impulsivity n (%)
Q10. The child tends to overlook details or make avoidable errors.	102 (60.71)	66 (39.29)	82 (50.62)	80 (49.38)	3.408	0.064	184 (55.76)	146 (44.24)
Q11. The child often talks excessively without pausing.	79 (47.02)	89 (52.98)	91 (56.17)	71 (43.83)	2.763	0.096	170 (51.52)	160 (48.48)
Q12. The child struggles to maintain attention and focus.	114 (67.86)	54 (32.14)	96 (59.26)	66 (40.74)	2.634	0.104	210 (63.64)	120 (36.36)
Q13. The child frequently has trouble organizing tasks or activities.	97 (57.74)	71 (42.26)	81 (50.00)	81 (50.00)	1.987	0.158	178 (53.94)	152 (46.06)
Q14. The child often lacks attention to detail in everyday tasks.	91(54.17)	77 (45.83)	76 (46.91)	86 (53.09)	1.735	0.187	167 (50.61)	163 (49.39)
Q15. The child frequently engages in running or climbing in inappropriate settings.	87 (51.79)	81 (48.21)	97 (59.88)	65 (40.12)	2.188	0.139	184 (55.76)	146 (44.24)
Q16. External distractions easily divert the child’s attention.	110 (65.48)	58 (34.52)	88 (54.32)	74 (45.68)	4.276	0.038	198 (60.00)	132 (40.00)
Q17. The child seems uninterested in face-to-face communication.	86 (51.19)	82 (48.81)	74 (45.68)	88 (54.32)	1.003	0.316	160 (48.48)	170 (51.52)
Q18. The child often interrupts or responds before a question is completed.	73 (43.45)	95 (56.55)	93 (57.41)	69 (42.59)	6.424	0.011	166 (50.30)	164 (49.70)
Q19. The child often acts independently but without clear initiative.	92 (54.76)	76 (45.24)	101 (62.35)	61 (37.65)	1.953	0.162	193 (58.48)	137 (41.52)
Q20. The child finds it difficult to wait or be patient.	81 (48.21)	87 (51.79)	95 (58.64)	67 (41.36)	3.603	0.057	176 (53.33)	154 (46.67)

**Figure 3:**
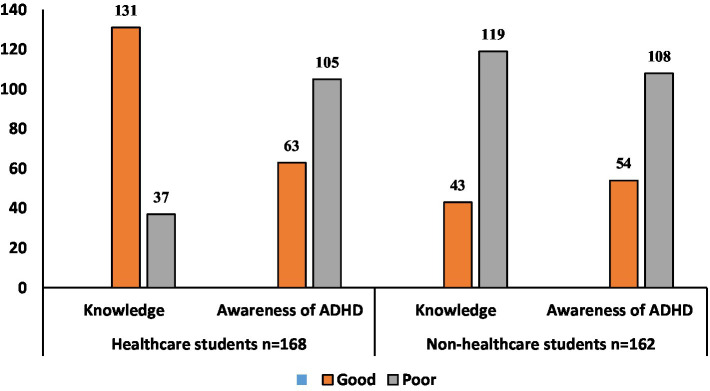
Categorization of knowledge and awareness scores related to ADHD among the study population.

Table 4 shows overall knowledge and awareness scores. Among healthcare students (*n* = 168), 131 (78.0%) had good knowledge, though only 63 (37.5%) had good awareness. Among non-healthcare students (*n* = 162), 43 (26.5%) had good knowledge and 54 (33.3%) had good awareness. Overall, 174 students (52.7%) had good knowledge, while only 117 (35.4%) demonstrated good awareness, highlighting substantial gaps across the student population (Figure 3).

**Table 4:** Descriptive statistics and categorization of knowledge and awareness scores related to ADHD among the study population.

Scores	Healthcare students		Non-healthcare students		Total population	
	Knowledge (%) (*n* = 168)	Awareness of ADHD (*n* = 168) (%)	Knowledge (%) (*n* = 162)	Awareness of ADHD (*n* = 162) (%)	Knowledge (%) (*n* = 330)	Awareness of ADHD (*n* = 330) (%)
Good	131 (78.0)	63 (37.5)	43 (26.5)	54 (33.3)	174 (52.7)	117 (35.4)
Poor	37 (22.0)	105 (62.5)	119 (73.5)	108 (66.7)	156 (47.27)	213 (64.5)

Multivariable logistic regression analysis indicated that GPA and source of information were most strongly associated with ADHD knowledge and awareness (Table 5). Among healthcare students, lower GPA (≤4.25) was associated with lower knowledge (OR = 0.48; 95% CI: 0.26–0.88; *p* = 0.019) and lower awareness (OR = 0.489; 95% CI: 0.302–0.793; *p* = 0.029), whereas reliance on healthcare providers for information was associated with higher knowledge (OR = 0.61; 95% CI: 0.38–0.98; *p* = 0.044). For non-healthcare students, lower GPA (≤4.25) was associated with lower knowledge (OR = 0.29; 95% CI: 0.13–0.66; *p* = 0.004) and lower awareness (OR = 0.354; 95% CI: 0.218–0.574; *p* = 0.006), while reliance on non-healthcare sources was associated with lower knowledge (OR = 0.55; 95% CI: 0.32–0.93; *p* = 0.029) and lower awareness (OR = 0.561; 95% CI: 0.349–0.900; *p* = 0.019). Other variables, including age, gender, academic year, and marital status, were not consistently associated with either outcome. These findings emphasize the associations between academic performance, sources of information, and students’ understanding of ADHD. Misconceptions about ADHD particularly the belief that any attention deficit equals ADHD and that it can be diagnosed via blood tests remain prevalent, even among healthcare students. These gaps highlight the need for targeted educational interventions to improve recognition, reduce stigma, and promote evidence-based understanding (Table 6).

**Table 5:** Multivariable logistic regression analysis of ADHD knowledge among healthcare and non-healthcare students.

Independent variables		Healthcare				Non-healthcare			
	Group	*B* value	*p*-value	OR	95% CI for OR	*B* value	*p*-value	OR	95% CI for OR
Age	5 years and above	Reference							
	18–20 years	0.812	0.048*	2.25	1.01–5.01	0.562	0.092	1.75	0.91–3.36
	21–24 years	0.678	0.072	1.97	0.94–4.10	0.302	0.231	1.35	0.83–2.41
Gender	Male	Reference							
	Female	0.523	0.036*	1.69	1.04–2.75	0.356	0.118	1.43	0.91–2.26
Year	Fifth Year	Reference							
	Second year	−0.785	0.042*	0.46	0.22–0.94	−0.442	0.160	0.64	0.34–1.23
	Third Year	−0.112	0.742	0.89	0.44–1.81	0.124	0.671	1.13	0.64–2.01
	Fourth Year	0.378	0.204	1.46	0.81–2.62	0.291	0.214	1.34	0.84–2.15
Marital status	Single	Reference							
	Married/Divorced	−0.176	0.692	0.84	0.35–2.01	−0.538	0.227	0.58	0.24–1.41
GPA	More than 4.75	Reference							
	>4.5 to 4.75	−0.213	0.424	0.81	0.47–1.38	−0.562	0.052	0.57	0.32–1.00
	>4.25 to 4.5	−0.598	0.026*	0.55	0.33–0.92	−0.765	0.013*	0.47	0.26–0.85
	Less than or equal to 4.25	−0.722	0.019*	0.48	0.26–0.88	−1.243	0.004*	0.29	0.13–0.66
Sources of information of ADHD	Healthcare providers	Reference							
	Other sources	−0.487	0.044*	0.61	0.38–0.98	−0.602	0.029*	0.55	0.32–0.93

B: Beta value; OR: Odds ratio; CI: Confidence Interval; *p < 0.05 considered statistically significant.

**Table 6:** Multivariable logistic regression analysis of ADHD awareness among healthcare and non-healthcare students.

Independent variables	Group	Healthcare				Non-healthcare			
		*B* value	*p* value	OR	95% CI for OR	*B* value	*p* value	OR	95% CI for OR
Age	25 years and above	Reference							
	18–20 years	0.476	0.112	1.610	0.986–2.630	0.289	0.317	1.335	0.818–2.179
	21–24 years	0.392	0.188	1.480	0.910–2.405	0.217	0.428	1.243	0.766–2.016
Gender	Male	Reference							
	Female	0.288	0.209	1.334	0.816–2.181	0.251	0.245	1.285	0.786–2.101
Year	Fifth Year	Reference							
	Second year	−0.423	0.072	0.655	0.402–1.068	−0.183	0.348	0.833	0.511–1.359
	Third Year	0.091	0.813	1.095	0.670–1.788	0.134	0.672	1.143	0.700–1.867
	Fourth Year	0.217	0.377	1.243	0.756–2.042	0.238	0.453	1.269	0.780–2.066
Marital status	Single	Reference							
	Married/Divorced	−0.134	0.715	0.875	0.526–1.455	−0.423	0.401	0.655	0.398–1.078
GPA	More than 4.75	Reference							
	>4.5 to 4.75	−0.321	0.093	0.725	0.448–1.174	−0.495	0.057	0.610	0.379–0.981
	>4.25 to 4.5	−0.581	0.041*	0.559	0.345–0.905	−0.698	0.035*	0.498	0.310–0.800
	Less than or equal to 4.25	−0.715	0.029*	0.489	0.302–0.793	−1.039	0.006*	0.354	0.218–0.574
Sources of information of ADHD	Healthcare providers	Reference							
	Other sources	−0.403	0.048*	0.668	0.415–1.077	−0.578	0.019*	0.561	0.349–0.900

B: Beta value; OR: Odds ratio; CI: Confidence Interval; *p < 0.05 considered statistically significant.

## 4 Discussion

The present study evaluated ADHD knowledge and awareness among university students in Saudi Arabia. Overall, 52.7% of students demonstrated good knowledge, while only 35.4% exhibited good awareness. Among healthcare students, 78.0% showed good knowledge compared to 26.5% of non-healthcare students, yet awareness remained low in both groups (37.5% vs. 33.3%). These findings align with previous studies reporting that 67 and 48.2% of healthcare students had a stronger theoretical understanding of mental health conditions such as ADHD (19, 23). Nonetheless, the low awareness even among those with good knowledge suggests a gap between academic knowledge and practical recognition, possibly due to limited clinical exposure or applied mental health education in early academic years (19, 28). This trend mirrors broader findings indicating that primary care professionals and medical trainees may possess theoretical knowledge but show limited applied awareness and confidence in identifying ADHD (29, 30).

The substantial gap in knowledge and awareness among non-healthcare students is particularly concerning. A 2023 UAE study of 406 students aged 18–20 found that non-healthcare students had significantly lower ADHD knowledge scores, with 34.7% exhibiting symptoms suggestive of probable ADHD (31). Similarly, in our study, healthcare students outperformed non-healthcare students in knowledge (78.0% vs. 26.5%), while awareness remained low in both groups (37.5% vs. 33.3%). These consistent findings across regional contexts highlight the urgent need for targeted educational interventions to improve ADHD literacy among non-healthcare students.

Multivariable logistic regression showed that GPA and source of information were associated with ADHD knowledge and awareness. Students with lower GPAs (≤4.25–4.5) tended to show lower knowledge than those with higher GPAs (>4.75), consistent with previous studies in which 68% scored ≥13/20; interns from medicine or pharmacy programs, GPAs ≥4.5, and SPPLE passers showed higher knowledge and awareness than their peers (27). The observed association between GPA and ADHD knowledge may reflect differences in academic engagement and cognitive skills, with students who had higher GPAs tending to demonstrate stronger study habits, motivation, and ability to process and retain complex information, which were associated with better understanding of neurodevelopmental disorders like ADHD. Higher academic performance was associated with broader competencies such as critical thinking and evidence appraisal skills essential for interpreting complex conditions (32–34). Although no significant gender differences were observed in this study, cultural, social, and educational factors may influence knowledge and awareness of ADHD, such as gender-specific exposure to mental health topics or classroom engagement (35, 36).

Regarding sources of information, healthcare students most often cited healthcare providers (32.7%) and academic sources—books/journals (23.8%)—alongside internet/social media (23.8%). Non-healthcare students relied mainly on internet/social media (48.1%), followed by family/friends (18.5%) and mass media (14.8%). These patterns mirror prior reports where 51.5% identified social media as their main source (37), and a Saudi study in which internet (49%) and social media (34%) dominated, with mass media secondary (25). Among teachers, books (32.8%) and websites (28.7%) were leading sources, with 76.7% attending ADHD training but only 40.4% feeling well informed (38). Across groups, digital and mass-media channels predominate, while healthcare-aligned populations more often use structured materials. Reliance on social media as a primary ADHD information source was associated with misconceptions about diagnosis, symptoms, and management (39, 40). Non-healthcare students are particularly affected, which was associated with differences in help-seeking behaviors, reinforce stigma, and hinder early symptom recognition. These findings emphasize the need for universities and educators to provide credible online resources and integrate digital literacy programs, including official social media campaigns and curated modules, to improve knowledge accuracy and awareness.

Interestingly, while healthcare students overall performed better in recognizing inattention ADHD symptoms, non-healthcare students demonstrated greater awareness of certain hyperactivity–impulsivity behaviors. A significantly higher proportion of non-healthcare students identified behaviors such as interrupting others or difficulty waiting their turn, possibly reflecting greater exposure to visible behavioral symptoms in social or everyday contexts or through media portrayals emphasizing externalizing behaviors over inattentive or internalized ones.

In the present study, healthcare students were more attuned to subtle, clinical presentations, showing higher awareness of inattention symptoms—such as “overlooking details” (60.7% vs. 50.6%) and “easily distracted” (65.5% vs. 54.3%; *p* = 0.038)—whereas non-healthcare students more frequently recognized hyperactivity–impulsivity behaviors, e.g., “interrupting before a question is completed” (57.4% vs. 43.5%; *p* = 0.011). These findings align with prior research. Among Saudi health interns, 68.2% demonstrated good ADHD knowledge, and 90.4% correctly rejected the misconception that ADHD can be diagnosed via a blood test, contrasting with 45.5% of our students who held this belief (27). In Bahrain, special education undergraduates with ADHD coursework scored 86.4% on awareness, compared to 39.7% among those without such coursework (41). Similarly, Thai pharmacy students identified executive-function difficulties in only 14.4% of at-risk participants, indicating persistent under-recognition of inattention features (42). Global surveys also suggest that general populations tend to identify overt symptoms but often misunderstand diagnostic processes, with ADHD prevalence estimates ranging from 5 to 8% (43).

A particularly concerning finding of our study was the widespread misconception that ADHD can be diagnosed through a blood test, reported by 45.5% of participants—higher among non-healthcare students (54.9%) than healthcare students (36.3%). Such beliefs were associated with delayed recognition and referral, as individuals or families who expect laboratory confirmation may be less likely to seek psychiatric or psychological evaluation, prolonging untreated symptoms and functional impairment. Framing ADHD as a disorder requiring a “blood marker” also reinforces stigma, potentially leading some to dismiss the diagnosis or attribute difficulties to poor parenting or personal failings. Alarmingly, over one-third of healthcare students endorsed this belief, suggesting that even future clinicians may unintentionally perpetuate misinformation, with implications for both individual care and broader public understanding. Misconceptions about ADHD diagnosis thus were associated with delayed intervention, societal attitudes, and public health. In contrast, studies among medical students at Qassim University reported that 96% correctly recognized that ADHD cannot be diagnosed via a blood test, with similar awareness observed among healthcare interns in other regional populations (around 90%) (19, 23). Similar to findings in mental health research, misconceptions and limited knowledge about ADHD were associated with reinforcement of stigma and hindered timely recognition and care (44). Educational interventions targeting healthcare students may improve understanding and reduce these barriers.

Beyond academic and informational factors, cultural perceptions in Saudi Arabia were associated with persistence of such misconceptions. ADHD and other mental health conditions are often associated with stigma or explained through non-medical attributions, such as poor parenting, lack of discipline, or even spiritual causes (45–47). These cultural beliefs were associated with reduced help-seeking, limited discussion of symptoms, and perpetuation of diagnostic misconceptions. Stigma may also explain why awareness remained low even among healthcare students with stronger theoretical knowledge, highlighting that broader societal views exert an influence beyond the classroom.

An important observation is the apparent disconnect between knowledge and awareness. While healthcare students had higher knowledge scores, their awareness levels were not proportionately better than those of non-healthcare students, highlighting the limitations of passive learning. This suggests the need to integrate interactive and experiential methods such as formal coursework, clinical simulations, case-based discussions, or exposure to real-life scenarios into curricula. Evidence from systematic reviews and pilot trials indicates that targeted online or case-based ADHD education can measurably improve knowledge and reduce misconceptions among healthcare providers (25, 48–50). Beyond regional surveys, recent international research has evaluated promising interventions to enhance ADHD literacy and awareness. A 2024 meta-analysis of gamified digital mental health interventions (DMHIs) reported significant improvements in ADHD symptoms among children and adolescents, highlighting the value of interactive, technology-based approaches to engagement and learning (51). Similarly, a 2024 scoping review of psychoeducational group interventions for adults with ADHD confirmed their feasibility, acceptability, and effectiveness in improving knowledge and self-management outcomes (52). In educational settings, an experiential “Active Awareness Training” model in Europe using simulation of ADHD-like difficulties followed by debriefing was associated with improved understanding and reduced stigma more effectively than traditional psychoeducation (44). Incorporating such innovative, evidence-based approaches into university curricula and awareness campaigns could help bridge the knowledge–awareness gap identified in this study.

Despite variations in knowledge and awareness levels, chi-square analysis revealed no significant differences between healthcare and non-healthcare students based on demographics such as age, gender, GPA category, marital status, or year of study. These results suggest that academic discipline and source of information are associated with differences in ADHD understanding rather than inherent demographic factors, consistent with other regional surveys (19, 23). Overall, these findings reflect differences in recognition of ADHD symptom domains—inattention and hyperactivity–impulsivity—between healthcare and non-healthcare students, highlighting the potential value of structured, evidence-based education. Targeted educational strategies such as formal coursework, case-based learning, and social media–based awareness campaigns could be considered to support improved conceptual clarity, reduce misconceptions, and facilitate balanced recognition of both inattention and hyperactivity–impulsivity symptoms.

### 4.1 Practical recommendations

Based on the study findings, several measures may help improve ADHD knowledge and awareness among university students. First, universities may integrate structured mental health education into curricula, emphasizing both inattention and hyperactive–impulsive symptom recognition. Interactive approaches such as clinical simulations, case-based discussions, and experiential learning might help bridge the gap between theoretical knowledge and applied awareness. Second, awareness campaigns through official social media platforms and curated digital modules may support access to credible ADHD information. Third, interdisciplinary workshops involving healthcare and non-healthcare students could contribute to broader understanding and help reduce stigma. Finally, faculty development and training on ADHD may encourage educators to provide accurate guidance and model best practices for mental health literacy.

### 4.2 Broader mental health implications

The gaps in ADHD knowledge and awareness observed in this study are associated with potential challenges for broader mental health understanding. Misconceptions about diagnosis and symptom recognition are linked to delayed intervention and may coincide with academic, occupational, and social difficulties. Reliance on inaccurate information sources is associated with lower help-seeking behaviors and under-recognition of ADHD. Among healthcare students, unaddressed misconceptions could be reflected in professional practice and broader societal understanding. These observations underline the importance of improving ADHD literacy for individual well-being and for fostering a community capable of recognizing, supporting, and appropriately managing neurodevelopmental conditions.

### 4.3 Limitations

This study has several limitations that should be considered when interpreting the results. First, the use of a non-probability stratified purposive sampling technique may limit the generalizability of the findings to the broader university student population, as participants were not randomly selected. This may have introduced selection bias, potentially overrepresenting students with greater interest or exposure to mental health topics. Stratification by faculty and year of study was applied to partially mitigate this concern and enhance subgroup representation. Although stratified purposive sampling ensured representation across healthcare and non-healthcare students and different academic years, the non-probability design limits generalizability. The findings are therefore representative of the sampled population only, and caution should be exercised when extending conclusions to students at other universities or regions. Second, a critical limitation is the gender imbalance, with 60% of participants being female, which restricts the ability to generalize findings equally across male and female students. Third, data were collected via a self-administered online questionnaire distributed through social media and institutional platforms, which may have introduced self-selection bias. Although English is the medium of instruction at the university, some students may have had limited proficiency. To improve comprehension, a pilot study with 60 students (30 healthcare, 30 non-healthcare) was conducted, leading to minor revisions for clarity and cultural appropriateness. While formal cognitive debriefing or full linguistic validation was not performed, the pilot ensured general understandability. The self-report nature of the survey may have further introduced response bias, potentially affecting the accuracy of reported knowledge and awareness. Future studies could consider randomized or incentivized sampling strategies and formal linguistic validation to enhance representativeness, reliability, and inclusivity.

Fourth, the cross-sectional design limits causal inferences, as associations between variables such as academic performance and ADHD knowledge were assessed at a single time point. Longitudinal research is recommended to examine changes over time, particularly in response to educational interventions or clinical exposure. Fifth, reliance on self-reported data may have introduced recall or social desirability bias, affecting the accuracy of reported knowledge and awareness. Incorporating objective assessments, such as knowledge quizzes or external evaluations, could enhance validity. Sixth, the study did not collect data on participants’ personal history of ADHD diagnosis or treatment, which may influence knowledge and awareness levels. Future research should consider including this information while safeguarding participants’ well-being and confidentiality. Lastly, the study was conducted at a single public university in Saudi Arabia, which may limit external validity. Multi-center studies involving diverse institutions and regions are recommended to allow broader generalizations and cross-cultural comparisons.

## 5 Conclusion

This study identified notable gaps in ADHD knowledge and awareness among university students, with healthcare students demonstrating higher theoretical knowledge than non-healthcare peers, although practical awareness remained relatively low in both groups. Associations were observed between higher GPA, access to professional or curriculum-based information, and academic discipline with better knowledge and awareness; however, these relationships should be interpreted as correlations rather than causal effects. Critical gaps persisted in misconceptions about diagnosis and under-recognition of inattentive symptoms. To address these issues, targeted strategies are recommended, including ADHD-focused workshops, case-based learning, and experiential simulations for healthcare students; interactive seminars, digital resources, and evidence-based campaigns for non-healthcare students; and dissemination of accurate information through official online platforms to reduce reliance on social media. These approaches aim to enhance ADHD literacy, support timely recognition, reduce stigma, and promote mental health awareness within university environments. Future research should examine the effectiveness of these strategies longitudinally and across multiple institutions to enable broader generalizations and cross-cultural comparisons. By promoting structured, evidence-based education and awareness programs, universities can provide students with knowledge, skills, and attitudes that may help them better understand and support individuals with ADHD and other mental health conditions.

## Data Availability

The original contributions presented in the study are included in the article/Supplementary material, further inquiries can be directed to the corresponding author/s.
