# Supplementary material for: Bridging the gap: a cross-sectional study on knowledge and awareness of attention-deficit/hyperactivity disorder among students at a public university
Source: Front Public Health. 2025 Sep 29;13:1679269. doi: 10.3389/fpubh.2025.1679269 (PMC12515941; doi:10.3389/fpubh.2025.1679269)
Supplement: Supplementary file 1 [file Data_Sheet_1.PDF]

Bridging the Gap: A Cross-Sectional Study on Knowledge and Awareness of Attention-Deficit/Hyperactivity Disorder Among Students at a Public University

Section 1: Sociodemographic Variables

Age
18-20 years
21–24 years
25 years and above
Gender
Male
Female
Department
Healthcare colleges
Non-healthcare colleges
Year of Education
Second year
Third Year
Fourth Year
Fifth Year
Marital Status
Single
Married/Divorced
GPA
More than 4.75
>4.5 to ≤ 4.75
>4.25 to ≤ 4.5
Less than or equal to 4.25
What are your sources of information about ADHD?
Healthcare providers
Through internet/social media
Family members/Friends
Television/Newspaper
Books/Journals/University studies

Section : 2 Knowledge About ADHD
Q1. Do you know what ADHD stands for or refers to?
Yes
No
Q2. How long must a patient show symptoms of ADHD before a diagnosis can be made?
One
Two
Six months
Twelve months
Q3. How many types does ADHD have?
One type
Two types
Three types

Q 4. ADHD is diagnosed in any child with an attention deficit.

Yes
No
Q5. Typically, earlier symptoms of ADHD are reported.
Home/ Parents
School/Teachers
Both
Q6. Does ADHD negatively impact a child's academic performance?
Yes
No
Q7. Is ADHD always associated with other neurological or psychiatric conditions?
Yes
No
Q8. Can ADHD be detected through a blood test?
Yes
No
Q9. Which approaches are used to treat ADHD?
Pharmacotherapy
Behavioral therapy
Both

Section 3: Awareness (Inattention and Hyperactivity–Impulsivity) Please indicate for each question whether it is Attention Deficit or Hyperactivity–Impulsivity.

Q10. The child tends to overlook details or make avoidable errors.
Q11. The child often talks excessively without pausing.
Q12. The child struggles to maintain attention and focus.
Q13. The child frequently has trouble organizing tasks or activities.
Q14. The child often lacks attention to detail in everyday tasks.
Q15. The child frequently engages in running or climbing in inappropriate settings.
Q16. External distractions easily divert the child’s attention.
Q17. The child seems uninterested in face-to-face communication.
Q18. The child often interrupts or responds before a question is completed.
Q19. The child often acts independently but without clear initiative.
Q20. The child finds it difficult to wait or be patient.
